# Periodontal disease: A systemic condition

**DOI:** 10.1111/prd.12616

**Published:** 2024-11-04

**Authors:** German E. M. Villoria, Ricardo G. Fischer, Eduardo M. B. Tinoco, Joerg Meyle, Bruno G. Loos

**Affiliations:** ^1^ Department of Periodontology, School of Dentistry Rio de Janeiro State University Rio de Janeiro Brazil; ^2^ Department of Periodontology, School of Dentistry Federal University of Rio de Janeiro Rio de Janeiro Brazil; ^3^ Dental School University of Berne Berne Switzerland; ^4^ Department of Periodontology, Academic Center for Dentistry Amsterdam (ACTA) University of Amsterdam and Vrije Universiteit Amsterdam Amsterdam The Netherlands

**Keywords:** comorbidity, multimorbidity, periodontal disease, periodontitis, systemic disease

## Abstract

For decades, periodontitis has been considered to be a local inflammatory disease of the periodontal tissues in the oral cavity. Initially, associations of periodontitis with a multitude of noncommunicable diseases were each studied separately, and relationships were shown. The associations of periodontitis with morbidities, such as cardiovascular diseases, rheumatoid arthritis, diabetes mellitus, respiratory diseases, have been demonstrated. As most such studies were cross‐sectional in nature, questions about causality cannot be univocally answered. And periodontitis as an independent risk factor for one systemic disease, becomes even more difficult to assess since recently periodontitis has also been associated with multimorbidity. Periodontitis and many systemic diseases share environmental, lifestyle and genetic risk factors, and share immunopathology. Moreover, suffering from one common noncommunicable disease may increase the susceptibility for another such chronic disease; the systemic effects of one condition may be one of various risk factors for another such disease. The overarching effect of any systemic disease is it causing a pro‐inflammatory state in the individual; this has also been shown for periodontitis. Moreover, in periodontitis a prothrombotic state and elevated immunological activity have been shown. As such, when we consider periodontal disease as another systemic disease, it can affect the susceptibility and progression of other systemic diseases, and importantly, vice versa. And with this, it is not surprising that periodontitis is associated with a variety of other noncommunicable diseases. The medical definition of a systemic disease includes diseases that affect different organs and systems. Thus, the aim of this opinion paper is to propose that periodontitis should be considered a systemic disease in its own right and that it affects the individual's systemic condition and wellbeing. The dental and medical profession and researchers alike, should adapt this paradigm shift, advancing periodontal disease out of its isolated anatomical location into the total of chronic noncommunicable diseases, being for some conditions a comorbid disease and, vice versa, comorbidities can affect initiation and progression of periodontal disease.

## 1 BACKGROUND AND AIM OF PAPER

Periodontal disease is one of the most common inflammatory diseases in humans. Periodontitis remains a worldwide public health problem, with a combined prevalence of almost 62% in dentate adults. For moderate‐to‐severe cases, the estimated combined prevalence is 53.2%, while for severe periodontitis it is 23.6%, according to studies carried out between 2011 and 2020.^1^ These results show a higher prevalence compared to estimates from 1990 to 2010, where the prevalence of severe periodontitis was estimated at 10.8%.^2^ Another study showed that the prevalence was substantially increased globally in the last three decades (up to 2019), now affecting 1.1 billion individuals.^3^ Periodontal disease is more common than cardiovascular disease, the leading cause of morbidity and mortality, which has a global prevalence of 6.6%.^4^

Periodontitis is also considered a global public health problem,^5^ not only affecting periodontal health but also having implications for the patient's general health and wellbeing.^6^ Because of this broad impact, it clearly needs increased recognition and attention for the overall health of mankind,^5^ and with this, health professionals themselves need to gain more awareness on periodontal disease and its effect on the overall health of their patients.

Traditionally, periodontitis was considered to be a local inflammatory disease of the periodontal tissues in the oral cavity. However, in the last four decades there has been growing awareness in the scientific community on the associations between periodontal disease and various other types of chronic systemic diseases and conditions.^7^ From the mid‐eighties of the previous century, associations of periodontitis with various noncommunicable diseases were each studied separately, and relationships were shown. Close to 30% of all periodontal research proposals in clinical research trial registries dealt with the association of periodontitis with other diseases.^8^ A recent bibliometric analysis^9^ on the relationship between periodontitis and cardiovascular diseases shows a growing trend in the last 30 years of the yearly number of publications on this topic. After 2017, the number of published articles on possible associations between periodontitis and cardiovascular diseases consistently reached the 60‐article threshold, with an average of approximately 77 articles per year. This trend reached its maximum with 97 articles in 2021. The associations of periodontitis with other morbidities and the plausible biological explanations have been shown in many studies; for example, for atherosclerotic cardiovascular diseases, rheumatoid arthritis, Alzheimer's disease, diabetes mellitus, obesity, and respiratory diseases.^7^, ^10^ As most such studies are cross‐sectional in nature, questions about periodontitis being causally related cannot be answered. And to establish whether periodontitis is an independent risk factor for one systemic disease, becomes even more difficult to assess since recently periodontitis has also been associated with multimorbidity;^11^, ^12^, ^13^ this may also explain residual confounding in most studies. Therefore, the complexity of which and how many chronic diseases co‐occur and interact with each other, grows as we know more.

Periodontitis and many systemic diseases share environmental, lifestyle and genetic risk factors, and share immunopathology.^14^, ^15^, ^16^, ^17^, ^18^, ^19^ It is clear that suffering from one common noncommunicable disease may increase the susceptibility for another such chronic disease; the systemic effects of one condition may be one of the various risk factors for another such disease. The overarching characteristic of any chronic noncommunicable systemic disease is it being associated with a pro‐inflammatory state in the individual^20^; this has also been shown for periodontitis and will be reviewed below. As such, when we consider periodontal disease as a systemic disease, it will affect the susceptibility and progression of another systemic disease, and importantly, vice versa. Therefore, the consistent findings of the associations of periodontal disease with multiple chronic diseases is not surprising.

The medical definition of a systemic disease includes diseases that affect different organs and systems.^21^, ^22^ Thus, the aim of this opinion paper is to propose that periodontitis should be considered a systemic disease in its own right and that it affects the individual's systemic condition and wellbeing. The dental and medical profession and researchers alike, should adopt this paradigm shift, advancing periodontal disease out of its isolated anatomical location into the total of chronic noncommunicable diseases, being for some conditions a comorbid disease and, vice versa: comorbidities can increase susceptibility and progression of periodontitis.

## 2 PERIODONTITIS IS A COMPLEX INFLAMMATORY DISEASE

Periodontitis is a complex, multicausal inflammatory disease. The various causal factors play a role simultaneously and interact, resulting in the individual's immunological dysregulation; moreover, a dysbiotic biofilm is another challenge to the host response having already an underlying dysregulated/maladaptive function and can in this way induce a vicious cycle.^16^, ^23^, ^24^, ^25^, ^26^ Both environmental factors and intrinsic factors play a role and are categorized within five clusters. Intrinsic causal factors are (epi)genetic risk factors; various genetic variants have been identified and validated in genome‐wide association studies (cluster 1). The environmental factors are the inflammation‐triggering microbial communities (cluster 2), unhealthy lifestyle factors (cluster 3), comorbidity(ies) and systemic conditions (cluster 4), and tooth and dentition‐related factors (cluster 5). For each patient, the relative contribution of the five clusters of causal factors varies and needs to be estimated.^16^ Also, age plays a role, as with increasing age susceptibility to any disease increases due immunosenescence.^27^, ^28^, ^29^, ^30^ Further, it is important to note that the effects of the unfavorable lifestyle habits such as smoking and unhealthy diet, on different people living in comparable environments, often differ substantially, indicating the important gene–environment interactions.

Periodontal disease shares common risk factors with other chronic diseases, such as genetic profiles, that is, the concept of pleiotropy.^14^, ^15^, ^16^, ^17^, ^18^, ^31^, ^32^ Concerning genetic risk factors, at least four genetic risk factors are shared between periodontitis and cardiovascular disease.^14^, ^18^, ^32^ Furthermore, periodontitis shares lifestyle risk factors (smoking, poor diet, stress) and unfavorable systemic conditions (obesity, diabetes)with, for example, cardiovascular diseases.^33^, ^34^, ^35^, ^36^, ^37^ Smoking, a high carbohydrate intake and fat‐rich diet, and a limited intake of fruit and vegetables are risk factors for cardiovascular diseases as much as they are for periodontitis.^38^, ^39^ Smoking is also a significant risk factor for rheumatoid arthritis,^40^ another inflammatory disease associated with periodontitis. Obesity is a shared risk factor between type 2 diabetes mellitus, cardiovascular diseases and periodontitis.^41^ Notably, the shared lifestyle risk factors and a (chronic) hyperglycemic state may also contribute to shared epigenetic changes accrued in life. With these examples, it is easy to explain why periodontitis is associated with many other diseases. The effects of shared risk factors on pathophysiological processes in many noncommunicable chronic diseases also suggest sharing various inflammatory pathways.

## 3 PRO‐INFLAMMATORY STATE, PRO‐THROMBOTIC STATE, AND OTHER SYSTEMIC EFFECTS

Although periodontitis is an inflammatory disease of the supporting tissues of the teeth, the effects of the inflammation are not compartmentalized. Through the circulation (blood, lymph) and through inhalation of bacterial pathogens, systemic effects can occur, either directly via leakage (transmigration) of microorganisms, microbial components, inflammatory mediators, and activated immune cells from the periodontal lesions, or indirectly, when other organs are responding with inflammatory or aberrant activity to the transmigrated mediators and microbial components.^42^ The increased response of the liver is such an example of the latter phenomenon, but also blood vessels themselves (the vascular system as organ) have locations (atherosclerotic lesions) where increased inflammatory activity is present due to the above‐described transmigrated components. Therefore, periodontitis can be labeled as a systemic disease by its ability to cause a pro‐inflammatory state and a prothrombotic state, and altered immunological activities; and the effects thereof on the various bodily organs.

### 3.1 Pro‐inflammatory state

#### 3.1.1 C‐reactive protein and fibrinogen

C‐reactive protein (CRP) and also fibrinogen are acute‐phase reactant proteins produced in response to infection and inflammation. They are synthesized mainly by hepatocytes in the liver in response to various pro‐inflammatory cytokines, notably IL‐6, and in response to bacterial components. CRP is used as a biomarker for the initial diagnosis of infectious and inflammatory diseases, as an indicator of the need to treat acute and chronic conditions and to monitor the clinical evolution of diseases such as cardiovascular disease, rheumatoid arthritis, metabolic syndrome, diabetes mellitus, obesity, and cancer.^43^ Traditionally, the systemic impact of bacterial infections has been monitored by CRP and fibrinogen.

A relatively large number of clinical cross‐sectional studies and periodontal treatment studies have been carried out to assess plasma/serum CRP, and plasma fibrinogen in peripheral blood in healthy individuals and patients with periodontitis.^18^, ^44^ For example, researchers observed that levels were increased in periodontitis patients when compared to healthy controls.^45^ Also, evidence has shown a positive relationship between increased CRP and the severity of periodontitis.^46^, ^47^ Further, studies have evaluated the effect of periodontal treatment and CRP levels. Evidence shows that nonsurgical periodontal therapy, whether performed intensively or conventionally, causes decreases in CRP, including a progressive reduction up to 6 months.^44^, ^48^, ^49^, ^50^ Other studies also show that high‐sensitivity C‐reactive protein levels decreased after periodontal treatment and that this reduction was significant in individuals with cardiovascular disease as a comorbid condition.^51^, ^52^ A recently published meta‐analysis confirms the reduction in CRP and IL‐6 after conventional periodontal therapy.^53^

Taken together, the inflammatory process in the periodontal tissues have their ramifications elsewhere such as the activation of liver tissue to produce elevated levels of CRP and fibrinogen. This is strong evidence for periodontitis being a systemic disease.

#### 3.1.2 Interleukins and TNF‐α

Among the many immunological mediators secreted during inflammatory processes, several pro‐inflammatory interleukins (IL‐1, IL‐6, IL‐8) and tumor necrosis factor‐alpha (TNF‐α) have been extensively studied for their possible systemic presence in periodontitis.^42^ Although other immune mediators (e.g., IL‐17, PGE2) have been suggested in elevated levels in serum and/or plasma of periodontitis patients,^54^, ^55^ we focus here on a limited number of immunological mediators that have been the subject of the most research and have a particularly strong pro‐inflammatory activity. The significant correlation found for specific cytokines present in plasma or serum and gingival crevicular fluid support the proposition that the inflammatory reactions in periodontitis are not restricted to the diseased sites in the mouth.^56^ Another systematic review with meta‐analysis showed that in patients with heart and kidney transplants, there was evidence that periodontitis is associated with higher serum levels of IL‐6 than those without periodontitis.^57^

These findings seem to be present in populations of various ethnic origins; study populations have originated from all over the world. For example, also increased serum levels of IL‐6 were found in American Indian/Alaskan natives with moderate chronic periodontitis compared to healthy individuals or those with mild disease, after controlling for possible confounding factors such as gender, body mass index, smoking, and high‐density lipoprotein.^58^

A case–control study was carried out to estimate circulating levels of TNF‐α in the saliva and serum of patients with chronic periodontitis and periodontally healthy individuals. In the absence of systemic diseases, an increase in TNF‐α levels in both fluids was demonstrated in chronic periodontitis compared to normal healthy patients.^59^

Studies have also shown that elevated systemic levels of pro‐inflammatory mediators can be reduced as a result of periodontal therapy, attesting to another level of evidence that the systemic increase of these mediators are indeed related to periodontitis. The effect of nonsurgical periodontal therapy showed a significant decrease in serum IL‐6 and IL‐8 levels.^60^, ^61^, ^62^ Furthermore, the results of a systematic review in which body mass index (BMI) was considered a confounding factor in sample selection, suggest that periodontal treatment reduces serum IL‐6 levels in individuals with type 2 diabetes.^63^ Interestingly, in individuals with stable coronary artery disease and with chronic periodontitis, nonsurgical periodontal therapy decreased serum levels of TNF‐α, IL‐6 and CRP, which could help to reduce the inflammatory burden in these individuals.^61^

#### 3.1.3 Other systemic markers associated with periodontitis

Matrix metalloproteinases, especially MMP‐8 and MMP‐9, degrade the extracellular matrix in the periodontium and regulate remodeling processes in cardiovascular diseases, especially in atherosclerotic plaques. Therefore, these biomarkers are valuable for periodontitis and an important measure of the systemic connection.^64^ Patients with chronic periodontitis have elevated serum levels of MMP‐8^65^ and MMP‐9^66^ compared to patients with gingivitis or healthy controls.

Adipokines (leptin, adiponectin, and chemerin) and resistin are a group of bioactive molecules secreted mainly by adipose tissues. They have been described due to their critical role in the immune response, bone and lipid metabolism, energy expenditure, and modulation of insulin sensitivity.^67^ A recent meta‐analysis demonstrated elevated serum levels of leptin (pro‐inflammatory) and decreased serum levels of adiponectin (anti‐inflammatory) in periodontitis patients compared to controls within the total of study populations having a BMI <30 kg/m^2^. Also, evidence has shown that patients with chronic periodontitis have higher serum resistin levels compared to healthy individuals.^68^, ^69^ A systematic review and meta‐analysis supported increased serum leptin levels and decreased serum adiponectin levels in periodontitis patients compared to controls in the BMI <30 kg/m^2^ population, and showed that in systemically healthy periodontitis patients, serum leptin and adiponectin levels did not change significantly after periodontal treatment.^70^ Further, in a study that aimed to quantify two adipokine molecules (ghrelin and chemerin) released in association with food intake and obesity in periodontally healthy and diseased individuals across different body mass categories, it was shown that both groups with chronic periodontitis had statistically significantly higher levels of chemerin in serum than the group of periodontally healthy/gingivitis individuals with BMI <25 kg/m^2^.^71^ Another study found that chemerin and inflammatory cytokines such as IL‐1β, IL‐6, and TNF‐α were notably higher in the serum of patients with chronic periodontitis than those in the control group, (periodontally healthy/gingivitis).^72^

Other less studied pro‐inflammatory mediators have also been demonstrated in the systemic circulation in periodontitis patients. For example, a cross‐sectional study aiming to investigate whether increasing periodontitis severity, bleeding and ulcerated periodontal inflamed surface area (PISA), showed that serum and salivary levels of neutrophil gelatinase‐associated lipocalin increased in proportion to the severity of the disease and the ulcerated periodontal inflamed surface area.^73^ To evaluate the contribution of chronic periodontitis to serum procalcitonin levels in patients with chronic migraine, a cross‐sectional study was carried out. It concluded that chronic periodontitis contributes to elevated serum procalcitonin levels in patients with chronic migraine, suggesting that chronic periodontitis may play a role in the chronification of migraine.^74^ In another case–control study published in 2019, the authors found that in the chronic migraine group, patients with periodontitis had higher serum levels of calcitonin gene‐related peptide and IL‐6, while nonsignificant differences were observed for IL‐10 concentrations compared to those without periodontitis.^75^

Compared to healthy controls, monocyte chemoattractant protein‐1 (MCP‐1) levels were statistically significantly higher in serum subjects with chronic periodontitis^76^, ^77^ and its levels decrease after periodontal therapy.^78^ MCP‐1/CC‐2 chemokine ligand (CCL2) is identified due to its monocyte chemotactic property in vitro in human cell lines. The role of MCP‐1 has been implicated in the pathogenesis of various diseases by numerous mechanisms, such as Alzheimer's disease, cardiovascular disease, rheumatoid arthritis, diabetes mellitus, and its complications.^79^

### 3.2 Pro‐thrombotic state

#### 3.2.1 Platelets

Platelet numbers and their activation state are crucial for the onset and exacerbation of thrombotic events and atherogenesis.^80^, ^81^ Evidence has shown that patients with periodontitis have elevated platelet activation compared to age‐ and sex‐matched controls.^82^ Subgingival debridement significantly decreases the gram‐negative anaerobic asaccharolytic species, such as *Porphyromonas gingivalis*, which show a strong association with platelet activation.^82^ A cross‐sectional study carried out on a representative sample of the South Korean population tested the presence of an association between periodontitis and platelet count. It showed that severe periodontitis is independently associated with a considerable increase in platelet count, which is explained, at least in part, by an increase in systemic inflammation.^83^

#### 3.2.2 Fibrinogen

Important molecular agents of the coagulation cascade, such as thrombin and fibrinogen, are epidemiologically and mechanically linked to diseases with an inflammatory component. The pro‐inflammatory function of fibrinogen has been reported in vascular wall diseases, stroke, multiple sclerosis, Alzheimer's disease, rheumatoid arthritis, colitis, pulmonary and renal fibrosis, and various types of cancer, among others.^84^ As was also discussed above, it is well documented that serum fibrinogen levels are higher in patients with periodontitis^45^, ^85^, ^86^ and that the treatment of periodontal disease reduces fibrinogen levels in these patients.^87^, ^88^

#### 3.2.3 Selectins

Selectins are an essential component of adhesion molecules and are cell membrane glycoproteins expressed on leukocytes, platelets and endothelial cells in the bloodstream that mediate the host's immune response.^89^ The primary ligand (PSGL‐1) on the surface of leukocytes interacts with selectin P, L, and E, each with varying affinity to mediate leukocyte adhesion and rolling on the endothelium and other signal transduction in the immune response to inflammation.^90^ In recent years, selectins and their ligands have been discovered as one of the essential factors connecting periodontal inflammation and the development of other systemic disorders, including chronic inflammatory diseases such as cardiovascular disease, diabetes, atherosclerosis, certain types of cancer and others.^89^ In a study by Avarnitidis et al., the authors observed that the expression of platelet membrane markers P‐selectin (an adhesion molecule) and CD63 (a molecule also involved in the regulation of cell adhesion and signal transduction) and the reactivity of platelets activated with the monoclonal antibody PAC‐1, were reduced after periodontal therapy, as was the propensity to form platelet‐leukocyte complexes, and consequently platelet hyperactivity. Therefore, periodontal treatment significantly decreases platelet activation in periodontitis and highlights the possible systemic benefit of this treatment.^80^, ^91^

#### 3.2.4 Von Willebrand factor and PAI‐1

Von Willebrand factor (VWF) is a large multimeric glycoprotein that plays an important role in hemostasis, illustrated by the bleeding tendency in von Willebrand disease, the most common inherited bleeding disorder caused by von Willebrand factor deficiency or dysfunction. VWF appears to be an essential participant in the multifaceted interaction between the hemostatic system and the atherosclerotic process.^92^ PAI‐1 is a protease inhibitor that decreases fibrinolysis by inhibiting tPA (tissue plasminogen activator) and uPA (urokinase). These properties are associated with an increased risk of atherosclerosis. In a study examining a variety of risk factors in patients with periodontitis and cardiovascular disease, significant but weak associations were reported between periodontal parameters and levels of von Willebrand factor and PAI‐1; however, follow‐up of the impact of nonsurgical periodontal therapy did not observe significant changes in levels of hemostatic factors, which may be due to pre‐existing cardiovascular disease and the difficulty of modifying these factors in these patients.^93^ A case–control study evaluated a series of thrombotic markers in patients with periodontitis, including PAI‐1, VWF, prothrombin cleavage fragments, and D‐dimer. They found that PAI‐1 was elevated in patients with advanced periodontitis compared to periodontally healthy individuals.^94^

#### 3.2.5 ICAM

Serum concentrations of soluble intercellular adhesion molecule‐1 (sICAM‐1) and soluble vascular cell adhesion molecule‐1 (sVCAM‐1) have been studied in various inflammatory diseases and systemic conditions, such as atherosclerosis, rheumatoid arthritis, systemic lupus erythematosus, and colorectal cancer, in which these cell adhesion molecules were detected at elevated levels compared to those in systemically healthy conditions.^95^ In an observational study comparing ICAM‐1, E‐Selectin and myeloperoxidase levels in individuals with moderate‐to‐severe chronic periodontitis and individuals with gingivitis or incipient periodontitis, data analysis showed significantly higher plasma levels of E‐selectin, myeloperoxidase (MPO), and ICAM‐1 in patients with moderate‐to‐severe periodontitis after adjusting for age and waist circumference.^96^

### 3.3 Elevated immune activity

In periodontitis, several immunological activities are affected. The immune response is generally heightened, and this includes both elevated and dysregulated activities. The pathophysiology of periodontitis involves an abnormal host response to the dysbiotic dental biofilm, with reduced or maladaptive immune capacity.^16^, ^25^, ^97^, ^98^ We summarized above the elevated pro‐inflammatory cytokines and the increased production of MMPs. However, also enhanced T‐cell and B‐cell activity is observed in periodontitis. If resolution of inflammation is not achieved, activation of T and B cells is critical in controlling chronic inflammation through constitutive cytokine secretion and modulation of osteoclastogenesis. T‐cells secrete cytokines that can exacerbate inflammation, while B‐cells produce antibodies that target periodontal pathogens.^99^ Also, elevated levels of immune complexes have been observed; in periodontitis, there is an increase in immune complexes consisting of antigens and antibodies that can further hyperactivate the immune response, both locally and in other organs.^97^, ^100^ Also, citrullination of proteins seems to be increased in periodontitis, which may be one of several explanations for the link between periodontitis and rheumatoid arthritis, atherosclerosis and Alzheimer's disease.^101^ Another example as was reviewed by Schenkein & Loos,^87^ patients with periodontitis have increased systemic antibody responses to periodontal microorganisms, and several of these organisms are capable of inducing specific and cross‐reacting antibodies relevant to atherosclerosis risk. These antibodies may, in turn, promote or influence systemic inflammatory responses and those within atheromatous lesions.^87^ Further, neutrophils show increased activity, including enhanced production of reactive oxygen species (ROS), elevated release of granular enzymes, and increased Neutrophil Extracellular Trap (NET)‐formation.^102^, ^103^ The formation of NETs has been associated with several diseases, including inflammatory diseases and autoimmune diseases such as rheumatoid arthritis.^103^ Nonsurgical periodontal therapy is able to restore the body's capacity to degrade NETs.^104^

### 3.4 Other systemic inflammatory indices being elevated in periodontitis

#### 3.4.1 Erythrocyte sedimentation rate

The erythrocyte sedimentation rate (ESR) is a commonly performed hematological test that can indicate and monitor an increase in inflammatory activity in the body caused by one or more conditions, such as autoimmune diseases, infections, or tumors. The ESR is not specific for any disease but is used with other tests to determine the presence of increased inflammatory activity.^105^ Two systematic reviews have recently been carried out to assess the effectiveness of nonsurgical periodontal therapy on rheumatoid arthritis disease‐activity. Nonsurgical periodontal treatment significantly reduced the Disease Activity Score and the ESR.^106^, ^107^ Similarly, another systematic review with meta‐analysis found significant reductions in the ESR and a trend toward a reduction in TNF‐α plasma levels after nonsurgical periodontal treatment,^108^ concluding that nonsurgical periodontal treatment can benefit patients with rheumatoid arthritis. These findings again attest to the general systemic effects and hematological effects of periodontitis.

#### 3.4.2 Cancer‐related systemic inflammatory indices in periodontitis

Clinicians in hospital settings dealing with systemic diseases and dealing with patients with several forms of cancer, often use systemic inflammation indices to indicate the extent of systemic involvement of the disease. These indices have not yet been extensively used to date in the field of periodontology; however, a recent review summarized several studies applying these systemic indices.^109^ Inflammation indices like the neutrophil to lymphocyte ratio, platelet‐to‐lymphocyte ratio, platelet distribution width, plateletcrit, red blood cell distribution width, lymphocyte‐to‐monocyte ratio, delta neutrophil index, and systemic immune inflammation index have been used in periodontitis by some investigators.

Among these indices elevated platelet distribution width (PDW) levels have been associated with certain types of cancer, that is, breast cancer, laryngeal cancer, and colorectal cancer. A meta‐analysis demonstrated strong evidence for high pre‐treatment PDW levels in association with poor advanced prognosis.^110^ The association between periodontitis and elevated PDW is corroborated by statistically significant correlations between PDW and periodontal inflamed surface area (PISA) (For further details, see the review by Walther et al. in this issue).^109^

For another marker, the systemic inflammation index (=SII), calculated as: (neutrophil count) × (platelet count)/(lymphocyte count), elevated levels have been observed in various kinds of cancer, reflecting a systemic inflammatory response and an imbalance in the immune system. Increased SII values have been associated with poor prognosis in several malignancies. Periodontitis can be regarded as a model of low‐grade systemic inflammation that exhibits a similar pathophysiology as in rheumatoid arthritis. SII may serve as a potential indicator of systemic inflammatory response; in periodontitis, a statistically significant association has been reported.^111^

Until today for the majority of cancer‐related systemic inflammatory indices, the level of evidence in relation to periodontitis is weak, which is due to the design of the studies and inconsistencies in the classification.

## 4 EXAMPLES OF SYSTEMIC DISEASES AS COMORBID DISEASES FOR OTHER SYSTEMIC DISEASES – A PARALLEL WITH PERIODONTITIS

One systemic disease can be a comorbidity for another systemic disease and as such could affect the other condition. We stated before that most noncommunicable diseases have an overarching commonality, that is, a pro‐inflammatory state, which could exacerbate and/or affect another chronic disease. To further show arguments that periodontitis is to be considered a systemic disease, we take here now an example on other systemic diseases, and how they are associated with and considered risk factors for other diseases.

In a recent single‐center cross‐sectional study, Beukers et al. investigated the prevalence of patterns of comorbidities and multimorbidities in patients with and without periodontal disease. The results of this study indicate that individuals with periodontal disease are more likely to be burdened by comorbidities and multimorbidities than those without any comorbidity (46.3% vs. 30.9% respectively; adjOR 1.36, CI 1.30–1.43). The presence of distinct clusters suggests a differential overlap in pathophysiology between periodontitis and certain systemic diseases. Periodontal disease can be considered part of multimorbidity as one systemic disease that occurs simultaneously in certain groups of individuals.^11^

The question is whether periodontitis, as a systemic disease, is analogous to other systemic diseases known to impact various organs and potentially exacerbate additional conditions, thereby serving as a risk factor for other diseases. Thus, can we draw comparisons with certain systemic diseases of the immune, digestive, or respiratory systems, which have been shown to influence other systems and organs, for example the cardiovascular system or kidneys. This potential interaction may also be applicable to periodontal disease, suggesting it could similarly affect multiple bodily systems and organs, thereby characterizing its systemic nature.

### 4.1 Rheumatoid arthritis

Rheumatoid arthritis is a chronic inflammatory disorder that primarily affects the joints. This autoimmune condition leads to joint inflammation characterized by pain, swelling, and stiffness. Over time, rheumatoid arthritis can result in joint destruction, deformity, disability, and even death.^112^ Beyond the joints, rheumatoid arthritis can also impact other parts of the body, including the skin, eyes, lungs, blood vessels, and heart; this systemic involvement makes rheumatoid arthritis another risk factor for cardiovascular disease.^113^ Additionally, rheumatoid arthritis can contribute to the development of interstitial lung disease, osteoporosis, and metabolic syndrome.^114^ Epidemiological studies suggest that synovial tissue and activated circulating immune cells in rheumatoid arthritis release pro‐inflammatory cytokines such as TNF‐α and IL‐6, which directly lead to systemic inflammation, a pro‐inflammatory state and the risk for cardiovascular disease.^115^, ^116^

Chronic inflammatory arthritis including rheumatoid arthritis, ankylosing spondylitis, and psoriatic arthritis are conditions at increased risk of morbidity and mortality for which a prognostic assessment is mandatory to support their effective clinical management. Interestingly, inflammation‐induced disease activity in rheumatoid arthritis patients is also related to metabolic syndrome, central obesity, dyslipidemia, and hypertension.^117^ Notably, metabolic syndrome is more frequent in the group of patients with rheumatoid arthritis, ankylosing spondylitis and psoriatic arthritis, being closely and directly related to the chronic systemic inflammation and disease activity. Also, chronic inflammatory arthritis, is associated with cancer, presence of moderate/high atherosclerotic disease activity, left ventricle structural and functional abnormalities, and is an independent prognosticator of adverse clinical events at mid‐term follow‐up. In light of these findings, an accurate assessment of metabolic syndrome should be routinely conducted in patients with rheumatoid arthritis, ankylosing spondylitis, and psoriatic arthritis as a measure of clinical outcomes which goes beyond the role of simple cardiovascular disease risk management.^117^

In sum, systemic effects of rheumatoid arthritis trigger a pro‐inflammatory state, and rheumatoid arthritis has been considered a risk factor for metabolic syndrome and cardiovascular disease. This state and being a risk factor for other diseases is also found for periodontitis as reviewed before. Therefore, based on the parallel with rheumatoid arthritis, also from that view point, periodontitis can be considered a systemic disease.

### 4.2 Inflammatory bowel diseases

Crohn's disease and ulcerative colitis are often combined into one group of inflammatory bowel disease (IBD). Crohn's disease and ulcerative colitis are systemic inflammatory diseases that primarily affect the gastrointestinal tract through a complex interaction of genetic background, environmental exposures (including the gut microbiome), and dysregulation of the innate and adaptive immune response.^118^, ^119^ However, inflammatory bowel disease not only affects the gastrointestinal tract but can also affect other organs and systems; as such inflammatory bowel disease has been identified as a risk factor for atherosclerotic CVD, respiratory disease, liver disease, and neurological conditions.^120^, ^121^ Further scientific reports have associated inflammatory bowel disease with an increased risk of for atherosclerotic CVD, heart failure, and atrial fibrillation.^122^, ^123^ Several inflammatory biomarkers, including CRP, serum amyloid, TNF‐α, interleukin IL‐6, IL‐8, IL‐17A, and calprotectin are significantly increased in inflammatory bowel disease patients.^124^ A study of over 100 000 individuals showed a significant increase in serum CRP levels in inflammatory bowel disease patients and with that an increased risk for atherosclerotic CVD.^125^ Elevated levels of the same pro‐inflammatory mediators in the circulation have been implicated in the association between periodontal disease and the development of atherosclerotic CVD.^18^, ^87^, ^126^, ^127^

Endocrine and metabolic manifestations of inflammatory bowel disease include metabolic bone disease, growth retardation, hypogonadism, pubertal delay, lipid abnormalities, and insulin resistance.^128^ The main explanation for the association between inflammatory bowel disease and diabetes is the chronic systemic inflammation, insulin resistance and intestinal dysbiosis.^129^, ^130^ In a large retrospective population‐based cohort study, the risk of type 1 and type 2 diabetes was significantly higher in patients with Crohn's disease, but not in those with ulcerative colitis after adjustment for steroid use and all diabetes risk factors, including age, sex, smoking, alcohol consumption, physical activity, body mass index, and baseline glucose levels. The researchers concluded that patients with Crohn's disease have a significantly increased risk of diabetes compared with controls without inflammatory bowel disease, regardless of steroid use.^131^ This resembles the association between periodontitis and diabetes. It is proposed that the systemic pro‐inflammatory state induced by periodontitis contributes to insulin resistance and, ultimately, to diabetic complications.^132^ Reducing systemic levels of pro‐inflammatory mediators through periodontal therapy, leading to decreased systemic inflammation, is probably in part responsible for controlling hyperglycemia.^133^, ^134^, ^135^

There is evidence of a bidirectional association whereby individuals with inflammatory bowel disease are at high risk of developing periodontal disease and vice versa; periodontal disease may influence the severity and activity of inflammatory bowel disease.^136^ This intricate association involves a complex pathogenic network influenced by immunological characteristics, microbial dysbiosis, and inflammatory cascades.^137^, ^138^, ^139^, ^140^

Overall, the systemic effects of Crohn's disease and ulcerative colitis induce a pro‐inflammatory state, and these diseases (inflammatory bowel diseases) are recognized as a risk factor for atherosclerotic CVD, diabetes, and some endocrine and metabolic manifestations. Similarly, periodontitis as a systemic disease, due to its induction of a pro‐inflammatory environment, can serve as a risk factor for other diseases.

### 4.3 Chronic obstructive pulmonary disease

The Global Initiative for Chronic Obstructive Lung Disease defines chronic obstructive pulmonary disease as “a heterogeneous lung disease characterized by chronic respiratory symptoms (dyspnea, cough, sputum production, and exacerbations) due to airway (bronchitis, bronchiolitis) and alveolar (emphysema) abnormalities causing persistent and often progressive airflow obstruction.”^141^ The pathogenesis of chronic obstructive pulmonary disease is complex and multifactorial, involving a combination of genetic and environmental factors, with cigarette smoke being the most widely accepted causal factor.^142^ The toxic chemicals in cigarette smoke result in chronic inflammation of the airways and lung parenchyma, causing the release of chemokines in these tissues and promoting the infiltration of neutrophils and other inflammatory cells into the airways.^143^, ^144^

A systematic review showed that people with chronic obstructive pulmonary disease have an increased risk for atherosclerotic CVD compared to the general population.^145^ Similarly, chronic obstructive pulmonary disease appears to be an independent risk factor for stroke, and this risk increases particularly during acute exacerbations of chronic obstructive pulmonary disease.^146^ The proposed mechanism is the release of pro‐inflammatory cytokines and inflammatory mediators, such as IL‐6 and TNF‐α, into the systemic circulation, causing systemic inflammation, which in turn may activate inflammatory cells in atherosclerotic plaques and can exacerbate the atherosclerotic lesion.^147^ These explanations are similar to the observed and reviewed systemic pro‐inflammatory mechanism for the link between periodontal disease and atherosclerotic CVD,^7^, ^18^, ^87^, ^126^ and once more shows that periodontitis as a systemic disease does not behave differently than other systemic diseases, including COPD.

## 5 EFFECTS OF SYSTEMIC DISEASES ON WELLBEING

### 5.1 Periodontitis

Quality of life (QoL) is defined by the World Health Organization as “an individual's perception of their position in life in the context of the culture and value systems in which they live and in relation to their goals, expectations, standards, and concerns,” and the same organization defines the term health as “a state of complete physical, mental, and social wellbeing and not merely the absence of disease.”^148^ Health‐related quality of life (HRQoL) is a multidimensional concept commonly used to examine the impact of one's health status on the individual's quality of life.^149^ An umbrella review of systematic reviews was conducted to analyze the broad impact of periodontal disease on overall HRQoL. The authors showed that periodontal disease can have a negative impact on an individual's systemic health and that both nonsurgical and surgical periodontal therapy can improve HRQoL, although to varying degrees depending on the patient's perception.^150^ Notably, the more severe the periodontitis, when symptoms such as bleeding, halitosis, and mobility are obviously present, the greater the negative impact on quality of life.^151^ In a study using the Oral Health Impact Profile‐14 questionnaire on more than 700 patients, those with more severe periodontal attachment loss rated their functional limitation, physical pain, psychological discomfort, and physical and psychological disability significantly worse than those with less severe disease.^152^

### 5.2 Rheumatoid arthritis

When we evaluate the impact of other systemic diseases on HRQoL, we see similar results as reported for periodontitis in terms of functional and psychological limitations. A systematic review and meta‐analysis showed that the impact of rheumatoid arthritis on HRQoL is substantial in both physical and mental domains. This supports recent current guidelines that state that patients with rheumatoid arthritis should be regularly assessed for the impact of their disease on HRQoL.^153^ A case–control study showed that the interaction effect of rheumatoid arthritis with PD had a significant impact on both, oral health‐related quality of life (OHRQoL) and HRQoL.^154^ For the management of rheumatoid arthritis access to a multidisciplinary team is considered essential for all aspects of wellbeing in relation to this disease^155^

### 5.3 Inflammatory bowel disease

Interestingly, the QoL of patients with inflammatory bowel disease deteriorates when simultaneously periodontal disease and other oral problems are present; a case–control study aimed to assess whether oral health problems affect quality of life in patients with inflammatory bowel disease and, conversely, whether inflammatory bowel disease affects OHQoL.^156^ The results of that study indicated that oral health problems, periodontal disease and tooth loss, are associated with poorer HRQoL specific to inflammatory bowel disease, and vice versa, inflammatory bowel disease is associated with poorer OHRQoL. These findings highlight the potential benefits of including dental professionals in multidisciplinary teams treating patients with inflammatory bowel disease.^156^

### 5.4 Chronic obstructive pulmonary disease

Studies have shown that poorer HRQoL was found in chronic obstructive pulmonary disease patients who had frequent exacerbations or multiple comorbidities.^157^ In addition to declining physical health, people with chronic obstructive pulmonary disease may experience anxiety and depression, which can have a significant impact on HRQoL.^158^ To maximize HRQoL in chronic obstructive pulmonary disease patients, also for this condition it was recommended to adopt a multidisciplinary approach consisting of different healthcare providers for maintenance of HRQoL while having to live with chronic obstructive pulmonary disease.^159^

### 5.5 Periodontitis and some common systemic conditions share reduced wellbeing

Similar effects of periodontal disease and other systemic diseases on individuals' HRQoL can be seen, affecting both physical and mental functioning. We found that periodontal disease can interact with other systemic diseases to worsen HRQoL and that there is a consensus that a multidisciplinary approach is considered essential for the assessment and management of aspects of wellbeing in relation to systemic diseases; inclusion of an oral health care professional in such teams seems obvious. Thus, we have further demonstrated that periodontitis should not be considered a local oral disease without any further effects on quality of life. Like other systemic diseases, periodontal disease has not only physiological measurable effects but also negative effects on wellbeing.

## 6 CONCLUSION

In this opinion paper, we propose that periodontitis should be considered as a systemic disease like many other chronic noncommunicable diseases. We took example of other systemic diseases; patients with these diseases have increased risks for atherosclerotic CVD, metabolic syndrome, diabetes, and other conditions. Periodontitis has comparable systemic effects as rheumatoid arthritis, inflammatory bowel disease, chronic obstructive pulmonary disease, and others. We reviewed literature that demonstrated a pro‐inflammatory state, a prothrombotic state and increased systemic inflammatory indexes in periodontitis that is very much alike these other well‐known systemic diseases. The pro‐inflammatory and prothrombotic states are risk factors for other morbidities. Also, we showed that periodontitis patients have a reduced health related wellbeing. Considering all the facts discussed above, we strongly encourage the dental and medical communities to embrace the understanding that periodontal disease extends beyond an isolated anatomical condition, as its systemic effects have been irrefutably proven. Recognizing periodontal disease as one that significantly impacts an individual's systemic condition and overall wellbeing is crucial. It is vital that healthcare professionals and policy makers collaborate to ensure that patients with periodontal disease receive comprehensive care, addressing both their oral health and its potential implications for other systemic conditions like it should be done when individuals suffer from rheumatoid arthritis, inflammatory bowel disease, chronic obstructive pulmonary disease, or other chronic diseases. This unified approach will enhance patient outcomes and promote overall health.

## CONFLICT OF INTEREST STATEMENT

The authors declare no conflict of interest, and this work was self‐funded.

## Data Availability

The data that support the findings of this study are available on request from the corresponding author. The data are not publicly available due to privacy or ethical restrictions.
